# Regulation of mononuclear phagocyte function by the microbiota at mucosal sites

**DOI:** 10.1111/imm.13155

**Published:** 2019-11-27

**Authors:** Nicholas A. Scott, Elizabeth R. Mann

**Affiliations:** ^1^ Lydia Becker Institute of Immunology and Inflammation University of Manchester Manchester UK; ^2^ Manchester Collaborative Centre for Inflammation Research Faculty of Biology, Medicine and Health Manchester Academic Health Science Centre University of Manchester Manchester UK

**Keywords:** dendritic cell, macrophage, microbiota, mucosal

## Abstract

Mucosal tissues contain distinct microbial communities that differ drastically depending on the barrier site, and as such, mucosal immune responses have evolved to be tailored specifically for their location. Whether protective or regulatory immune responses against invading pathogens or the commensal microbiota occur is controlled by local mononuclear phagocytes (MNPs). Comprising macrophages and dendritic cells (DCs), the functions of these cells are highly dependent on the local environment. For example, the intestine contains the greatest bacterial load of any site in the body, and hence, intestinal MNPs are hyporesponsive to bacterial stimulation. This is thought to be one of the major mechanisms by which harmful immune responses directed against the trillions of harmless bacteria that line the gut lumen are avoided. Regulation of MNP function by the microbiota has been characterized in the most depth in the intestine but there are several mucosal sites that also contain their own microbiota. In this review, we present an overview of how MNP function is regulated by the microbiota at mucosal sites, highlighting recent novel pathways by which this occurs in the intestine, and new studies elucidating these interactions at mucosal sites that have been characterized in less depth, including the urogenital tract.

## Graphical abstract

This review focuses on interactions between the microbiota and mononuclear phagocytes at mucosal sites. These include the intestine, lungs, oral cavity, bladder/urinary tract and reproductive tract.

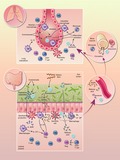

**Abbreviations**
AHR: aryl hydrocarbon receptor
AM: alveolar macrophage
BM: bone marrow
CCL: C‐C chemokine ligand
CCR: C‐C chemokine receptor
cDC: conventional dendritic cell
CSF‐1: colony‐stimulating factor 1
CXCL2: chemokine (C‐X‐C motif) ligand 2
DC: dendritic cell
GPR: G‐protein‐coupled receptor
IBD: inflammatory bowel disease
IL‐10: interleukin‐10
IM: interstitial macrophage
LPS: lipopolysaccharide
MLN: mesenteric lymph nodes
MNP: mononuclear phagocyte
RA: retinoic acid
RALDH1: retinaldehyde dehydrogenase 1
SCFA: short‐chain fatty acid
TGF‐*β*: transforming growth factor *β*
TLR: Toll‐like receptor
TNF‐*α*, tumour necrosis factor‐α
Treg cell: regulatory T cell
UTI: urinary tract infection

## Introduction

Mucosal immune responses are highly specialized to co‐exist alongside local microbial communities residing in mucosal tissues without provoking inappropriate inflammation, while preventing potential pathogens from crossing mucosal barriers. The local microbiota directly contribute to shaping these immune responses, and bacterial‐derived metabolites, both locally derived and from distal sites, play a key role in regulating mucosal immune function. Hence, each tissue has a highly specialized and specific immune system, with mononuclear phagocytes (MNPs), including dendritic cells (DCs) and macrophages, expressing pattern recognition receptors to recognize and respond to local microbiota constituents. Although MNP function has been characterized extensively in the intestine and lungs, recent evidence highlights novel pathways by which the microbiota regulate MNP function at these sites, which will be discussed in this review. Furthermore, there are various other mucosal sites that contain distinct microbial communities, including the oral cavity and urogenital tract. However, interactions between the microbiota and immune systems at these sites are poorly defined. This review will focus on the interactions between the microbiota and MNPs from the intestine, lungs, oral cavity, bladder/urinary tract and reproductive tract.

## Intestine

The intestinal immune system maintains the delicate balance between tolerance to harmless antigens, including the trillions of harmless bacteria comprising the commensal microbiota, and protective immunity against invading pathogens. When this balance is disrupted, inappropriate immune responses directed against the microbiota occur, which can lead to inflammatory bowel disease (IBD).^1^ The bacterial load in the intestine is greater than in any other part of the body, with trillions of bacteria accounting for a total weight of 1–2 kg.^2^ Hence, immune responses in the intestine are highly specialized and have adapted to co‐exist with this huge antigenic load. Intestinal MNPs, including DCs and macrophages, are essential for both immune tolerance and protective immunity in the intestine but these cells perform distinct functions and are differentially modulated by the microbiota to perform these roles. Although the intestinal microbiota predominantly comprises bacteria, interactions between intestinal fungi, viruses and MNPs directly modulate MNP function (reviewed in refs 3,4). However, given the vast amount of bacteria in the intestine combined with the large amount of information regarding bacterial modulation of intestinal MNP function, this section will focus on bacteria and their associated metabolites in tailoring intestinal immunity.

### Intestinal dendritic cells

Dendritic cells in the gut are highly specialized in their ability to generate intestinal T‐cell responses in mice^5^ and humans,^6^ and act as immune sentinels, constantly sampling antigen and constitutively migrating to the mesenteric lymph nodes (MLNs) in the steady‐state.^7^ This process is involved in the induction of regulatory T (Treg) cell responses directed against specific components of the microbiota to prevent overactive immune responses, and DC migration to the MLNs is increased upon Toll‐like receptor (TLR) stimulation.^8^ There are various subsets of intestinal DCs that can be distinguished based upon expression of CD103 and CD11b/Sirp*α* in mice^7^ and CD103 and Sirp*α* in humans^9^ with all of these subsets migrating in lymph towards the MLNs and capable of generating Treg cells *in vitro*.^10^ The conservation in the expression of CD103 and Sirp*α* on DCs across mice and humans make these useful markers to identify and compare DC subsets across species.

**Figure 1:**
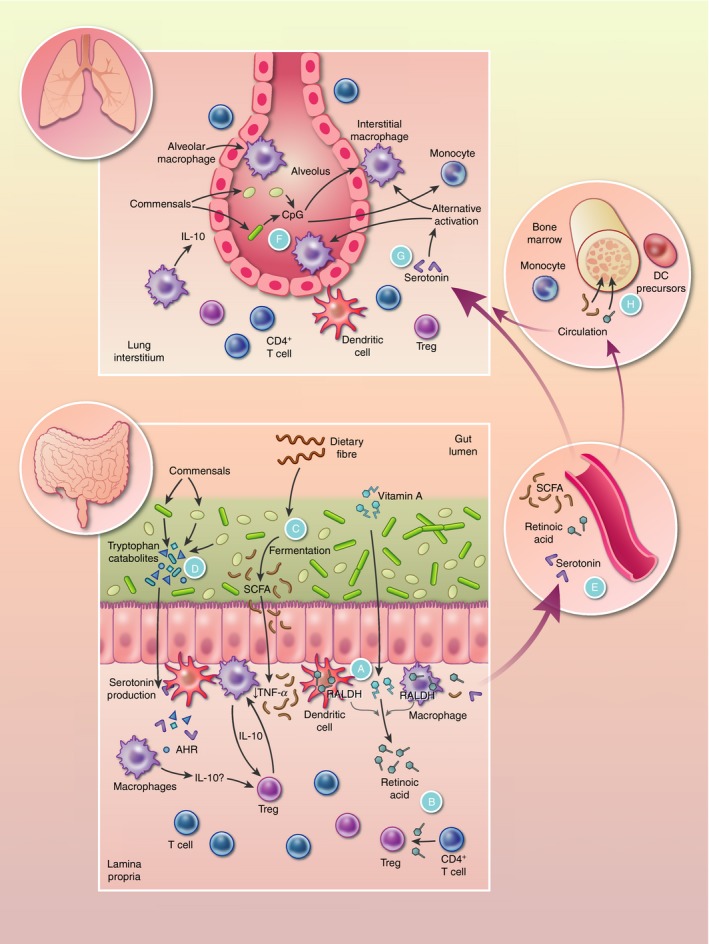
The commensal microbiome in the intestine and lung regulates immune responses both locally and at distal sites. Immune responses at mucosal sites are highly specialized to prevent inappropriate inflammation towards the local microbiota while also ensuring pathogens cannot cross mucosal barriers. Each tissue has dedicated mononuclear phagocytes (MNPs) comprised of macrophages or dendritic cells (DCs) located close to the epithelium whose function is influenced by commensals. Within the intestine, many of the dietary factors the microbiome processes alter MNP responses. (A) Dietary vitamin A is converted into retinoic acid by macrophages, DCs and intestinal epithelial cells (IECs), a process that has been demonstrated to rely on commensal support (see text). (B) Retinoic acid produced in this way by DCs is crucial for the induction of T regulatory (Treg) cells to induce tolerance to the microbiome. (C) The fermentation of dietary fibre into short‐chain fatty acids (SCFA) largely by Bacteroidetes and Firmicutes, ligates to G‐protein‐coupled receptors on macrophages and DCs or binds to histone deacetylases (HDACs) to promote tolerogenic responses. (D) Aryl hydrocarbon receptor (AHR) ligands, including tryptophan metabolites such as serotonin, are produced by microbes and recognized by AHR expressed by MNPs. (E) Many intestinal metabolites circulate systemically and localize to distal sites like the lungs and bone marrow (BM). (F) In the lungs, bacterial‐derived CpG can alter monocyte and interstitial macrophage function. (G) Serotonin may play a role in asthma development by enhancing alternative activation of monocytes and pulmonary macrophages. (H) SCFAs promote the production of Ly6C^−^ monocytes in the BM and retinoic acid affects the maturation of DC precursors.

The functions of intestinal DCs are in part shaped by the intestinal microbiota and its associated products (Fig. 1). For example, DCs in the intestine can retain small numbers of commensal bacteria for a number of days, which allows them to selectively induce IgA to help protect against mucosal penetration by commensals.^11^ Commensal bacteria resident in lymphoid tissue can induce production of the immunoregulatory cytokine interleukin‐10 (IL‐10) by intestinal DCs^12^ which also contributes to tissue‐protective functions in the context of intestinal barrier damage. Metabolites generated by the commensal microbiota in the intestine including short‐chain fatty acids (SCFAs) can shape DC development from precursors in the bone marrow (BM)^13^ and DC function.^14^, ^15^ Additionally, microbially derived SCFAs act on intestinal epithelial cells to enhance activity of the vitamin A‐converting enzyme retinaldehyde dehydrogenase 1 (RALDH1) in intestinal DCs^16^ (A in Fig. 1). Conversion of vitamin A to retinoic acid (RA) by intestinal DCs has previously been shown to be essential for the ability of intestinal DCs to generate gut‐homing Treg cells^17^, ^18^ (B in Fig. 1). Furthermore, RA also mediates the maturation of CD103^+^ CD11b^+^ DCs (cDC2) within the intestine^19^ and the generation of gut‐tropic migratory CD103^+^ DC precursors in the BM.^20^ However, many studies investigating the role of RA have relied on a diet deficient in vitamin A, which causes systemic inflammation, and therefore results should be interpreted with caution.

Transforming growth factor‐*β* (TGF‐*β*) is indispensable for generation of Treg cells in the intestine by CD103^+^ DCs,^17^ with activation of TGF‐*β* by intestinal DCs specifically being required for Treg cell generation and prevention of colitis.^21^ Interactions between the microbiota and intestinal DCs are likely to modulate these events given the direct effects of bacterially derived lipopolysaccharide (LPS) on the induction of the TGF‐*β*‐activating integrin *α*_v_/*β*_8_ by DCs.^22^ Integrin *α*_v_/*β*_8_ is predominantly expressed by CD103^+^ CD11b^−^ DCs in the intestine (cDC1)^23^, but probably tailors cross‐talk between DC subsets in the intestine as TGF‐*β* plays a fundamental role in the development and Treg‐generating function of CD103^+^ CD11b^+^ intestinal DCs (cDC2).^24^

Hence, evidence indicates that the overall effects of the microbiota and its products on intestinal DCs enhance their tolerogenic function. Indeed, human DCs in the colon exhibit enhanced regulatory properties compared with their counterparts from the small intestine^25^ corresponding with the enhanced microbial load in the colon.^26^ However, given the limited alterations in intestinal DCs from gnotobiotic (germ‐free) or antibiotic‐treated mice,^27^ the rest of this section will focus on macrophages.

### Origins and functions of macrophages in the intestine

The majority of macrophages in the intestine are seeded by Ly6C^hi^ blood monocytes in a largely CCR2‐dependent manner.^28^ However, recent evidence in mice indicates that a separate population of CCR2‐independent intestinal macrophages exists in the gut; these are long‐lived cells that express the markers Tim4 and CD4.^29^ These macrophages are present perinatally and play an essential role in intestinal physiology.^30^ Although definitive markers of intestinal macrophage turnover in humans have not been found, a similar hierarchy of subsets may exist in humans.^31^ Homing of monocytes to the intestine has been proposed to be driven by inflammatory antigens originating from the diet, infection or the commensal microbiota but requirements differ depending on the setting. For example, germ‐free mice have a reduced colonic macrophage compartment, which may result from diminished monocyte recruitment,^28^, ^32^ whereas small intestinal macrophage homeostasis appears dependent on dietary amino acids.^33^

The immunosuppressive cytokine IL‐10 plays a key role in the regulatory functions of intestinal macrophages. Genetic mutations in IL‐10 and the IL‐10 receptor in humans cause early‐onset IBD in children^34^, ^35^ and mice deficient in IL‐10 develop spontaneous colitis,^36^ indicating the critical role for IL‐10 in intestinal immune tolerance. Several immune cells in the intestine produce IL‐10, including Treg cells, epithelial cells, DCs and macrophages, and it was originally thought that commensal induced IL‐10 production by macrophages was critical for preventing colitis in mice.^37^, ^38^ However, it was since demonstrated that IL‐10 sensing by CX3CR1^+^ cells in the intestine (predominantly macrophages) is critical for the prevention of colitis, whereas IL‐10 production is redundant.^39^ As with global IL‐10‐deficient mice, inflammation and colitis in mice lacking the IL‐10 receptor on MNPs only developed in the presence of *Helicobacter *spp.,^40^ indicating a critical role for the microbiota in modulating macrophage‐mediated inflammation in the intestine. Interestingly, it was recently reported that attachment of commensal strains to the intestinal epithelium helps to support IL‐10 production by CX3CR1^+^ MNPs.^41^

### Compartmentalization of macrophage function in the intestine

The gastrointestinal tract differs significantly in function between locales. The small intestine is responsible for digestion and absorption of nutrients including protein, monosaccharides and vitamins. In contrast, one of the primary functions of the colon is the absorption of water. There are stark differences in oxygen content and pH levels between compartments, with hypoxia increasing and acidity decreasing moving from the small intestine to the colon.^42^ The mucus layer is much thicker in the colon than the small intestine, supporting the establishment of a much greater microbial load in the colon (reviewed in ref. 26). Indeed, the microbial load is many magnitudes higher in the colon (>10^11^ cells/g) compared with the small intestine (between 10^3^ and 10^8^ cells/g).^43^, ^44^ Anaerobic bacteria within the Bacteroidetes and Firmicutes phyla dominate the colon whereas the small intestine has an even ratio of aerobic and anaerobic bacteria.^45^ These different microbial and environmental conditions within these separate compartments of the intestine have led to the evolution of varying functions and roles for MNPs at these sites.

In the colon, high levels of SCFAs are generated by the fermentation of dietary fibre by several phyla of anaerobic bacteria (C in Fig. 1) including Bacteroidetes and Firmicutes that dominate the large intestine. Three SCFAs predominate in the intestine, acetate, propionate and butyrate, which are found at high physiological concentrations of 20–140 mm in mice and humans.^46^, ^47^ SCFAs have a profound effect on mucosal and systemic immune responses, with their importance indicated by abnormally low levels of SCFAs in the intestine being directly associated with several inflammatory diseases, including IBD (reviewed in ref. 48). Dietary plant fibres are a main energy source for bacteria that produce SCFAs^46^ and SCFA generated from the breakdown of dietary fibre contributes to the interplay between the microbiota, food intake and the immune system. Butyrate in particular has a wide range of potent immunoregulatory effects in the intestine^47^ and acts on intestinal macrophages to limit pro‐inflammatory cytokine production in response to bacterial stimulation. These effects of butyrate on macrophages were facilitated through inhibition of histone deacetylase activity^49^ and provide a mechanism for the phenomenon that intestinal macrophages are characteristically hyporesponsive to bacterial stimulation. This refractory response is defined by limited LPS‐induced production of inflammatory cytokines including tumour necrosis factor‐*α* (TNF‐*α*), IL‐6 and IL‐12.^50^, ^51^, ^52^

These hyporesponsive properties of intestinal macrophages are thought to be a key contributor to the prevention of overactive harmful immune responses against the microbiota that occur during IBD. Indeed, in humans, intestinal macrophages from the inflamed mucosa in IBD produce excess pro‐inflammatory cytokines in response to LPS^53^, ^54^ and low butyrate levels are found in the intestine in human IBD.^55^ Butyrate is recognized by cell surface expression of G‐protein‐coupled receptors including GPR109a, which is essential for the anti‐inflammatory effects of butyrate on intestinal macrophages.^56^ More recent studies in mice demonstrate that microbial disruption by antibiotic use drastically reduces SCFA levels in the intestine, with butyrate remaining low even during re‐population of the microbiota.^57^ These changes in SCFA levels corresponded with aberrant macrophage functions upon re‐population of the microbiota, with excess production of pro‐inflammatory cytokines leading to a long‐term induction of pro‐inflammatory T helper type 1 responses.^57^ Critically, supplementation of antibiotics with butyrate prevented immune dysfunction and butyrate directly induced an alternative activation signature in macrophages, with an increase in the expression of genes classically associated with alternative activation (*Arg1* and *retnla)* alongside increased expression of genes involved in oxidative phosphorylation.^57^ Additionally, another novel role for butyrate in macrophage function was recently demonstrated, showing that butyrate enhanced anti‐microbial peptide production from macrophages and protected against colitis‐causing intestinal infections. These effects were also mediated by alterations in macrophage metabolism, through inhibition of histone deacetylase 3 to inhibit mammalian target of rapamycin signalling and glycolysis.^58^ These two studies demonstrate a novel role for butyrate in shaping macrophage metabolism in the intestine, which has a profound effect on macrophage function.^59^

The small intestine contains high concentrations of dietary vitamin A and its active metabolite RA, an important modulator of intestinal immune responses. Vitamin A is converted to RA in a two‐step enzymatic process, first requiring alcohol dehydrogenases to generate the retinal intermediate followed by the need for RALDHs to generate RA.^60^ The expression of RALDH in the intestine is restricted to epithelial cells, macrophages and DCs, and hence these cells are crucial in controlling the availability of RA. Metabolized RA is sensed by ligation of retinoic acid receptors, nuclear receptors that control downstream transcription of RA target genes.^61^ The commensal microbiota acts on intestinal macrophages to produce the cytokine IL‐1*β*, which in turn induces intestinal innate lymphoid type 3 cells to produce granulocyte–macrophage colony‐stimulating factor and IL‐4, increasing RALDH expression and RA release by macrophages.^62^ These data indicate that the microbiota can indirectly shape RA signalling in macrophages. Although the effects of DC generation of RA in lymphoid tissue are well known,^17^, ^18^ the influence of macrophage generation of RA either directly or indirectly are less clear. Although local CX3CR1^+^ macrophages are critical for the stabilization of local Treg cell responses in the lamina propria,^63^ whether this is dependent on their ability to generate RA is not clear. Furthermore, in humans both small intestinal and colonic macrophages from IBD patients with Crohn's disease demonstrate increased expression and enzymatic activity of the RALDH isoform *ALDH1A1* compared with healthy controls, correlating with the increased differentiation of TNF‐*α*‐producing inflammatory macrophages.^64^ Hence, although RA has critical immunoregulatory properties, including generation of Treg cells, the capacity of macrophages to generate RA during inflammation appears to be increased. Although this may represent a feedback mechanism in response to inflammation, extrapolation of data obtained from murine studies in this case should be exercised with caution.

Another key dietary and microbial effector pathway involving intestinal macrophages is mediated by the aryl hydrocarbon receptor (AHR), which plays a critical role in regulating intestinal immunity.^65^ The AHR is a transcription factor sensor activated by a range of ligands including indoles and indole‐derived tryptophan metabolites from the microbiome or diet^66^, ^67^ (D in Fig. 1). Indole and tryptophan are used by many different components of the microbiota as an energy source, releasing catabolized derivatives into the local environment. For this reason, the intestinal microbiota is a source of AHR ligands, reflected by the fact that germ‐free mice or antibiotic‐treated mice have low levels of the AHR.^66^ However, as dietary ligands are also a major source of AHR ligands, immune regulation in both the small intestine and colon are likely to be at least partially dependent on AHR signalling. CD11c‐specific deletion of AHR in mice enhanced susceptibility to colitis^68^ although it is not known to what extent these effects were mediated by DCs versus macrophages because CD11c is expressed by both these cell types in the intestine. AHR signalling via signal transducer and activator of transcription 3 activation has been shown to promote IL‐10 expression in murine macrophage cell lines,^69^ but it is currently unclear whether this pathway is relevant to intestinal macrophages *in vivo*. (Table 1).

**Table 1:** Source and effects of metabolites on the intestinal and pulmonary immune systems

Metabolite	Source	Role of host/microbiome?	Effects	How sensed	References
Retinoic acid	Dietary vitamin A	Processed from vitamin A by MNPs and IECs with microbial assistance	Intestine: increase in Treg induction; T‐cell and B‐cell recruitment	Retinoic acid receptors	Coombes *et al*.;^17^ Denning *et al*.;^52^ Sun *et al*.;^18^ + Ref Mortha *et al*.^62^
AHR ligands	Microbiome; diet	Microbial catabolism of tryptophan and indoles	Intestine: loss of AHR on CD11c‐expressing cells increases DSS‐induced colitis; Promotion of macrophage IL‐10 production (cell lines)	Aryl hydrocarbon receptor	Chng *et al*.;^68^ Goudot *et al*.;^142^ Zhu *et al*.^69^
Secondary bile acids	Microbiome	Microbiota metabolism of liver‐derived primary bile acids	Intestine: tolerogenic properties (unclear mechanism)	GPBAR1 and FXR	Cipriani *et al*.;^143^ Vavassori *et al*.;^144^
Butyrate	Microbiome	Fermentation of dietary fibre (Bacteroidetes and Firmicutes)	Intestine: inhibits IL‐6, IL‐12 and NO in colonic macrophages Sustains production of IL‐10 by MNPs and Treg numbers Lungs: promotes alternative activation of lung macrophages through expansion of monocytes in the bone marrow	GPR41, GPR43 and GPR109a; inhibition of histone deacetylases; shifting metabolism directly?	Chang *et al*.;^49^ Schulthess *et al*.^58^ Scott *et al*.;^57^ Singh *et al*.;^56^ Trompette *et al*.^85^
Propionate	Microbiome	Fermentation of dietary fibre (Bacteroidetes and Firmicutes)	Lungs: alters DC precursors in the bone marrow, resulting in DC‐mediated suppression of Th2‐induced airway allergy	GPR41, GPR43; inhibition of histone deacetylases	Trompette *et al*.^84^
Serotonin	Host	Produced by Enterochromaffin cells, mast cells and myenteric neurons, promoted by the microbiota	Lungs: alveolar macrophages: Blocks IL‐12 and TNF‐*α* Increases IL‐10 and prostaglandin E_2_	Concentration regulated by serotonin transporter	Castro *et al*.;^90^ Gershon and Tack;^87^ Ménard *et al*.;^86^ Yano *et al*.^88^
Bacterial LPS and CpG	Microbiome	Lung microbiome and environmental	Lungs: reduces DC‐driven allergic Th2 responses (LPS) and expands IL‐10‐producing interstitial macrophages (CpG) Reduction in airway allergy	TLR4 and TLR9	Bedoret *et al*.;^79^ Sabatel *et al*.^81^

Abbreviations: AHR, aryl hydrocarbon receptor; DC, dendritic cell; DSS, dextran sodium sulfate; IEC, intestinal epithelial cells; IL‐10, interleukin‐10; LPS, lipopolysaccharide; MNP, mononuclear phagocytes; Th2, T helper type 2; TNF‐*α*, tumour necrosis factor‐*α*; Treg, regulatory T.

## Lungs

The lungs are continuously exposed to environmental challenges on a daily basis including inhaled microbes, but also contain their own distinct microbiota (reviewed in ref. 70). In health, it remains unclear to what extent the airways are stably colonized or whether the microbiota is constantly being replenished, but the composition of the lung microbiota changes with age and health status.^71^ Information regarding the role of the lung microbiota in shaping pulmonary immune responses is limited; however, there is emerging evidence for the functional impact of microbes in the airways on pulmonary immune homeostasis. Furthermore, bacteria‐derived metabolites originating from the intestine that enter the circulation (E in Fig. 1) have a profound effect on immune responses in the lung and in particular on MNP function. Although the lungs are continually exposed to inhaled microbes, including fungal spores, most information regarding fungal interactions with MNPs in the lung applies to the context of immune dysfunction and fungal disease/overgrowth rather than during homeostasis. The same applies for virus–MNP interactions during infection in the lung. Therefore, this section will focus on the immunomodulatory effects of commensal bacteria and their associated metabolites on MNPs in the lung.

### Pulmonary macrophages

The composition of the microbiota in the lower airways has been linked to pulmonary macrophage function, with genes associated with inflammation and remodelling expressed by macrophages during dysbiosis in the lung.^72^ Macrophages in the lung comprise distinct populations including alveolar macrophages (AMs) that reside in the airways and interstitial macrophages (IMs) that reside in the interstitial tissue.^73^ These macrophage populations have distinct origins and functions but the location of AMs in the airways enables them to be one of the first sites of contact with inhaled and local microbial species. As such, AMs express a broad range of cell surface receptors to enable them to sense local environmental signals including commensal microbiota or invading pathogens, which can cause inflammatory or suppressive signalling cascades depending on the type of stimuli.^74^

The effects of commensal bacteria in the lung on macrophage function are indicated by studies showing that disruption of commensal bacterial communities in the lung with antibiotic use lowered the frequency and number of AMs. These changes occurred alongside enhanced expression of genes encoding cytokines (*il13, il10, il6, il1β)* and alternative activation marker *Arg1*.^75^ Evidence also indicates that commensal bacteria in the respiratory tracts may act on macrophages to protect against viral infection; in this study bacterial colonization of *Staphylococcus aureus* recruited peripheral monocytes into the lung before their differentiation into macrophages exhibiting features of alternative activation, alongside a reduction in inflammation following influenza virus infection.^76^ Critically, macrophage function may be permanently altered following certain infections and exposure to microbial pathogens, with altered expression of TLRs^77^ alongside transcriptional and epigenetic reprogramming to enable heightened immune responses for subsequent infections.^78^

Interstitial macrophages in the lung interstitium are found in mice^79^and humans^80^ and spontaneously produce the immunoregulatory cytokine IL‐10. Although it has not been fully defined how IMs may directly interact with the local microbiota to potentially shape their function, exposure of IMs to low doses of LPS enables them to counteract the capacity of LPS‐activated DCs to induce Th2 responses against harmless allergens.^79^ Furthermore, IMs express a range of TLRs. Exposure to bacterial CpG expanded these regulatory IMs from monocytes (F in Fig. 1) either directly infiltrating the lung or after mobilization from the spleen, and these cells protected against allergy challenge.^81^ Hence, effects of the microbiota and its associated products on IMs in the lung may underlie the reduced risk of asthma development that is associated with exposure to microbes.^82^, ^83^

Metabolites from the gut microbiota can also shape immune responses in the lung (Fig. 1), which is the basis of the protective effects of a high‐fibre diet, which increases circulating SCFAs to protect against T helper type 2‐mediated allergic airway inflammation.^84^ In addition, these circulating microbial‐sourced SCFAs cause expansion of Ly6C^−^ monocytes in the BM, while concurrently enhancing expression of CD206 and programmed death protein ligand 1 (markers of alternative activation) by macrophages in the lung.^85^ This increased number of alternatively activated macrophages resulted in a decrease in the neutrophil chemoattractant CXCL1, blunting airway neutrophilia during influenza infection.^85^ The metabolite serotonin, whose production in the gut is promoted by the microbiome, is a neurotransmitter well known for its role in depression and anxiety. However, serotonin also modulates immune responses, and in particular inhibits the release of the pro‐inflammatory cytokines IL‐12 and TNF‐*α* while increasing production of anti‐inflammatory IL‐10 and prostaglandin E_2_ from AMs.^86^ More than 90% of the body's serotonin originates from the intestine, where it is synthesized by enterochromaffin cells as well as mucosal mast cells and myenteric neurons.^87^ Critically, serotonin synthesis in the intestine is promoted by the microbiota and its associated metabolites,^88^ and circulating platelets sequester serotonin from the gastrointestinal tract for distribution to various extraintestinal sites^89^ (E in Fig. 1). As such, serotonin is found in the lung where levels are regulated by the serotonin transporter on the lung endothelium.^90^ Further studies are needed to decipher the role of serotonin on pulmonary macrophage function *in vivo* although serotonin receptor agonists prevent the development of allergic asthma in mice^91^ (G in Fig. 1).

### Pulmonary DCs

The lung contains three major subsets of DCs including plasmacytoid DCs that are located in the airways,^92^ and two subsets of conventional DCs: CD103^+^ CD11b^−^ DCs (cDC1) and CD103^−^ CD11b^+^ DCs.^93^, ^94^ CD103^+^ cDC1 are located in the epithelial layer and can capture antigens directly from the airway lumen before migration to local lymph nodes,^95^, ^96^ whereas CD11b^+^ DCs reside in the lamina propria with no direct access to the airway lumen.^94^ Although all DC subsets play a pivotal role in anti‐viral immunity in the lung in the context of infection,^95^ there is arguably more of a role for CD103^+^ and plasmacytoid DCs in terms of conditioning by local commensal microbiota given their direct exposure to antigens in the airspaces. However, the effects of the local pulmonary microbiota on DC function during homeostasis have not yet been fully elucidated. Administered microbial stimuli signal via TLRs in both CD103^+^ and CD11b^+^ lung DCs to induce TGF‐*β* production, subsequently enhancing IgA class‐switch recombination in B cells and RA‐dependent induction of gut‐homing molecules integrin *α*_4_/*β*_7_ and CCR9 on B cells, enabling B‐cell migration to the intestine.^97^ It is unclear from this study what the direct effects of the local commensal microbiota in the respiratory tract are in this process. Immune crosstalk between the gut and the lung is bi‐directional, with bacteria‐derived metabolites originating from the intestine, including the SCFA propionate, act on DC precursors in the BM (H in Fig. 1), which go on to seed the lung, influencing the function of recently recruited DCs in the lung following allergy challenge. In this case, these newly recruited DCs suppress T helper type 2 cell proliferation and function, which is thought to contribute to the protective effects of propionate during allergy challenge in the lung.^84^

## Oral cavity

The oral cavity contains diverse microbial communities that alter significantly from other mucosal sites, comprising Firmicutes, Proteobacteria, Actinobacteria and Bacteroidetes with over 600 prevalent taxa present.^98^, ^99^ However, the role of the microbiota and its associated metabolites on immune regulation in the oral cavity and in particular on MNP function is not well characterized. The mononuclear phagocyte compartment contains macrophages, recruited monocytes^100^ and DCs^101^ although the origins and functions of these cells have not yet fully been elucidated. In particular, the link between recruited monocytes and macrophages is unclear.^102^ However, recruited monocytes can give rise to CD11b^+^ Langerin^+^ cells in the gingiva that share transcriptional properties with skin‐resident Langerhans cells.^103^ Studies involving gnotobiotic (germ‐free) mice indicate that microbiota‐independent mechanisms exist to support adaptive immunity, with gingiva‐resident T helper type 17 cell development being dependent on physiological mechanical damage that occurs during mastication (chewing).^104^ However, although recruitment of neutrophils to the gingiva can occur in the absence of the microbiota, commensal bacteria up‐regulate CXCL2 expression correlating with increased neutrophil recruitment, in a TLR‐dependent fashion.^105^ The experiments demonstrate that several microbiota‐independent mechanisms exist to shape immunity in the oral cavity but that arguably, innate immune responses may be partially dependent on the microbiota. Further studies are, however, critical to define the role of the oral and distal microbiota in shaping MNP function.

## Bladder and urinary tract

Although initially considered sterile, evidence now indicates the presence of commensal microbial communities in the bladder.^106^ However, how this commensal microbiota may shape immune function in the bladder and urinary tract has not been established. Many studies regarding bladder immunity are in the context of urinary tract infection (UTI). The persistence of UTIs in the absence of antibiotics and high incidence of recurrent UTIs in women^107^, ^108^ indicates that the local immune system and microbiota are unable to control potentially pathogenic bacteria entering the urinary tract and bladder. In other mucosal tissues, MNPs including DCs and macrophages play crucial roles in this form of immunosurveillance, and are major contributors of tissue homeostasis and defence.^109^ However, the functions of MNPs in the bladder and urinary tract and how they are modulated by the commensal microbiota are poorly understood. The importance of the local commensal microbiota is indicated by intentional colonization of the bladder of patients with recurrent UTIs (bacterial interference) as a means of preventing repeated infection.^110^ Evidence indicates that the composition of the vaginal microbiota also plays a role in the propensity to develop recurrent UTIs,^111^ suggesting distal effects of microbiota at other mucosal sites on bladder immune responses.

Macrophages are resident in the lamina propria of the bladder and notably express a variety of TLRs.^112^ Although interactions between the commensal microbiota and bladder macrophages have not been characterized, during UTIs bladder macrophages produce pro‐inflammatory cytokines following bacterial recognition by way of inflammasome activation.^113^ Resident macrophages in the bladder recruit Ly6C^+^ cells (and neutrophils) to the bladder from the bloodstream during infection. These recruited Ly6C^+^ cells expressed the macrophage marker F4/80 in the bladder tissue, indicating that these cells may represent an intermediary monocyte–macrophage population similar to that seen in the intestine.^50^ These recruited intermediary Ly6C^+^ cells secreted TNF, causing resident macrophages to secrete CXCL2, which in turn induces neutrophil production of matrix metalloproteinase‐9, needed for neutrophils to break through the basement membrane barrier to reach the epithelium.^114^ Therefore, a critical role of monocytes and macrophages in the bladder appears to be to regulate the functional activity of recruited neutrophils during infection. The contribution of recruited monocytes to the macrophage pool in the bladder during homeostasis is less clear. Although macrophages play a pivotal role in clearing infections in other mucosal tissues, resident bladder macrophages appear to restrict the development of adaptive immune responses following infection. These cells are highly efficient at bacterial uptake and successfully compete with local DCs, but result in poor T‐cell‐mediated immunity due to their inefficiency at antigen presentation.^115^

## Reproductive tract

The vaginal microbiota is largely dominated by bacteria (*Lactobacillus* spp.) but comprises fungi and viruses also,^116^, ^117^, ^118^ with all three playing important roles in reproductive health.^117^, ^118^, ^119^ The composition of the vaginal microbiota is heavily dependent on age, stage of menstrual cycle, hormonal fluctuations, sexual behaviour, and also the use of probiotics and antibiotics.^120^, ^121^, ^122^, ^123^ Like the bladder, historically the uterus was assumed to be sterile but it has since been demonstrated that human endometrial (uterine lining) samples contain distinct bacterial communities with *Lactobacillus* again being the most prevalent. Interestingly, in about one‐fifth of women, the bacterial community in the endometrium varied drastically from that in the vagina.^124^, ^125^ The association between changes in the reproductive tract microbiota and inflammatory disease strongly indicates that microbial–immune crosstalk is important for reproductive health. Indeed, endometrial dysbiosis can directly cause inflammatory diseases including chronic endometritis^126^, ^127^ whereas pathogenic bacteria are more prevalent in women with endometriosis^128^, ^129^. However, MNPs in the reproductive tract and interactions with the local microbiota to shape immune function in health and disease are extremely poorly characterized. Nonetheless, these cells are likely to play a critical role in tissue homeostasis and mediate inflammatory processes that shape disease outcome as they do in other mucosal tissues.

Endometrial (uterine) DC and macrophage numbers fluctuate throughout the menstrual cycle^130^ but most studies on endometrial MNPs have focused on macrophages. Endometrial macrophages vary in function throughout the menstrual cycle, and express pro‐inflammatory mediators including colony‐stimulating factor 1 (CSF‐1), macrophage inflammatory protein‐1*β* and macrophage migration inhibitory factor in the second half of the cycle, which is thought to play a role in preparing the endometrium for implantation. Macrophages in the endometrium also express matrix metalloproteinases during menstruation, which are pivotal for the breakdown of endometrial tissue, indicating a critical role for endometrial macrophages in tissue remodelling through the menstrual cycle. Depending on the stage of the cycle, endometrial macrophages exhibit a pro‐ or anti‐inflammatory phenotype (reviewed in ref. 131), probably correlating with their varying functions during the dynamic tissue remodelling that occurs throughout the menstrual cycle. Indeed, in a simulated menstruation model in mice, CSF1R^+^ monocytes and macrophages increase in number during repair and are localized to areas of breakdown, repair and remodelling in the endometrium,^132^ supporting a role for different populations of monocytes and macrophages in tissue remodelling and repair during homeostasis. It is unclear whether the increased numbers of endometrial monocytes during repair correlates to enhanced recruitment from the bloodstream. However, the dynamic expression of chemoattractant CCL2 by endometrial stromal cells under hormonal control throughout the cycle^133^ indicates that differential recruitment of CCR2^+^ monocytes into the uterus may occur during the menstrual cycle under the control of stromal cells. Further studies are critical to ascertain the fate of these monocytes and how this process may alter the functions of the endometrial macrophage pool. It is not known how endometrial macrophages respond to the endometrial commensal microbiota. However, the direct role of the microbiota in diseases such as chronic endometritis^126^, ^127^ alongside the microbial‐sensing functions of macrophages and their expansion in chronic endometritis^134^ strongly suggest that macrophage–microbial crosstalk contributes to inflammation and homeostasis in the uterus.

Macrophages and DCs are abundant in the vaginal lamina propria^135^ with DC characterization mainly being in the context of inflammation or infection, during which monocyte‐derived DCs and plasmacytoid DCs increase in number, derived from recruited precursors from the bloodstream.^136^, ^137^ Both types of MNPs in the vagina play an important role in host defence, especially in the context of sexually transmitted infections (reviewed in ref. 138) but these cells have not been characterized in depth and information regarding modulation of MNP function by the commensal microbiota is scarce. Dysbiosis that occurs in the vagina during bacterial vaginosis includes the expansion of several types of anaerobic bacteria and *Gardnerella vaginalis*.^139^, ^140^ Despite the protective effects of SCFAs at other mucosal sites for inflammation and infections, these anaerobic bacteria produce SCFAs during bacterial vaginosis, which are abundant in the genital tract. Interestingly, butyrate, propionate and acetate levels are increased further in vaginal fluid of women with bacterial vaginosis.^141^ However, whether these increased levels of SCFA contribute to inflammation and their effects on the local immune system is not clear.

## Conclusions/perspective

Local and distal microbiota have distinct functional effects on mucosal macrophages and DCs to modulate their function, but the functions of these cells differ drastically depending on their tissue of residence. The local tissue microenvironment is critical in shaping MNP function in addition to the microbiota, with specific factors including local cytokines or dietary‐derived metabolites acting alone or in concert with the microbiota to tailor MNP function at mucosal sites. Further investigations are required to fully understand how the MNP system is regulated by the microbiota at different mucosal sites. In particular, understanding the differential effects of local commensal microbiota versus effects of microbial products that access mucosal tissues (or BM) through the circulation will allow the potential for various therapeutic options by manipulating the microbiota. The oral cavity and urogenital tract are mucosal sites where the in‐depth functions of MNPs and how these are shaped by the microbiota are not known yet, despite the direct roles of these cells in response to infections and inflammatory and reproductive diseases. As such, it is difficult to draw parallels between all of the mucosal sites discussed, for example, whether SCFAs have differential effects on mucosal macrophages from different tissues. Nonetheless, the effects of SCFAs on MNP precursors in the BM with the potential to seed various mucosal tissues indicate that bacteria‐derived metabolites originating from the intestine may not only influence functions of MNPs directly but may also have indirect effects on MNP fate in a wide range of tissues. In light of the increasing prevalence of immune‐mediated disorders that have been associated with dysbiosis, modulating the microbiota will allow the potential to prevent disease and may contribute to the maintenance of remission in immune‐mediated disorders.

## Disclosure

There are no competing interests from either author.
